# Supplementary material for: δ-Lactones—A New Class of Compounds That Are Toxic to E. coli K12 and R2–R4 Strains
Source: Materials (Basel). 2021 May 30;14(11):2956. doi: 10.3390/ma14112956 (PMC8199173; doi:10.3390/ma14112956)
Supplement: Supplementary file 1 [file materials-14-02956-s001.zip › materials-1201923-supplementary.pdf]

materials

Supplementary Material

δ-Lactones—A New Class of Compounds That Are Toxic to *E. coli* K12 and R2–R4 Strains

Paweł Kowalczyk [1,*], Gawdzik Barbara [2,*], Damian Trzepizur [3], Mateusz Szymczak [4], Grzegorz Skiba [1], Stanisława Raj [1], Karol Kramkowski [5], Rafał Lizut [6] and Ryszard Ostaszewski [3]

1 Department of Animal Nutrition, The Kielanowski Institute of Animal Physiology and Nutrition, Polish Academy of Sciences, 05-110 Jabłonna, Poland; g.skiba@ifzz.pl (G.S.); s.raj@ifzz.pl (S.R.)
2 Institute of Chemistry, Jan Kochanowski University, Swietokrzyska 15 G, 25-406 Kielce, Poland
3 Institute of Organic Chemistry PAS, Kasprzaka 44/52, 01-224 Warsaw, Poland; damian.trzepizur@icho.edu.pl
4 Department of Molecular Virology, Institute of Microbiology, Faculty of Biology, University of Warsaw, Miecznikowa 1, 02-096 Warsaw, Poland; mszymczak@biol.uw.edu.pl
5 Department of Physical Chemistry, Medical University of Bialystok, Kilińskiego 1 str, 15-089 Bialystok, Poland; kkramk@wp.pl
6 The John Paul II Catholic University of Lublin, Institute of Mathematics, Informatics and Landscape Architecture ul. Konstantynów 1 H, 20-708 Lublin, Poland; lizut@kul.pl
* Correspondence: p.kowalczyk@ifzz.pl (P.K.); b.gawdzik@ujk.edu.pl (G.B.)

Citation: Kowalczyk, P.; Trzepizur, D.; Szymczak, M.; Skiba, G.; Kramkowski, K.; Ostaszewski, R. δ-Lactones—A New Class of Compounds That are Toxic to *E. coli* K12 and R2–R4 Strains. *Materials* **2021**, *14*, 2956. https://doi.org/10.3390/ma14112956

Academic Editors: Francesco Baino and John T. Kiwi

Received: 13 April 2021
Accepted: 27 May 2021
Published: 30 May 2021

Publisher's Note: MDPI stays neutral with regard to jurisdictional claims in published maps and institutional affiliations.

Copyright: © 2021 by the authors. Licensee MDPI, Basel, Switzerland. This article is an open access article distributed under the terms and conditions of the Creative Commons Attribution (CC BY) license (https:// creativecommons.org/licenses/by/ 4.0/).

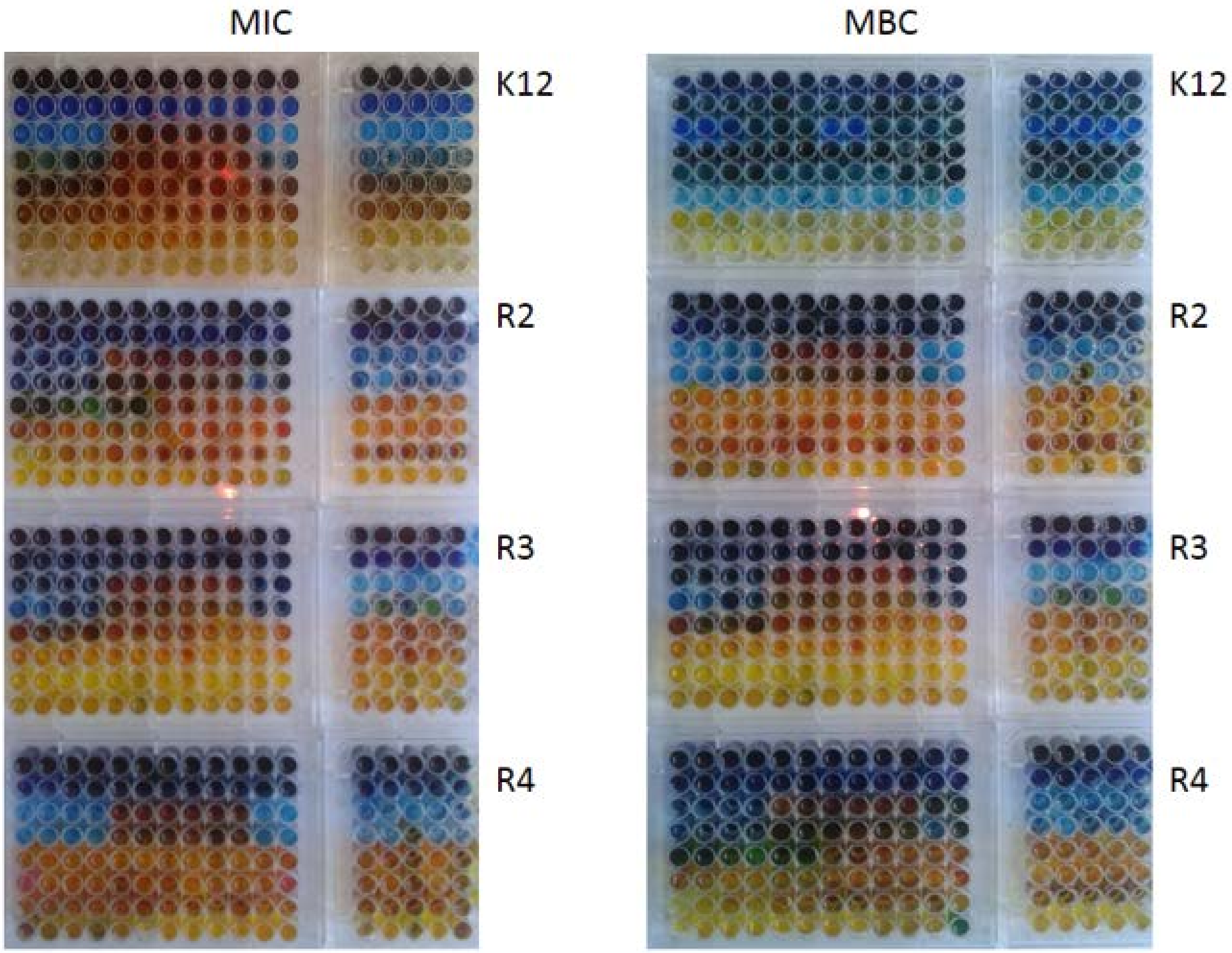

Figure S1. Examples of MIC and MBC on microplates with different concentration of studied compounds (mg L−1). Resazurin was added as an indicator of microbial growth with K12, R2, R3 and R4 strains with tested 17 compounds. as described in Table 1.

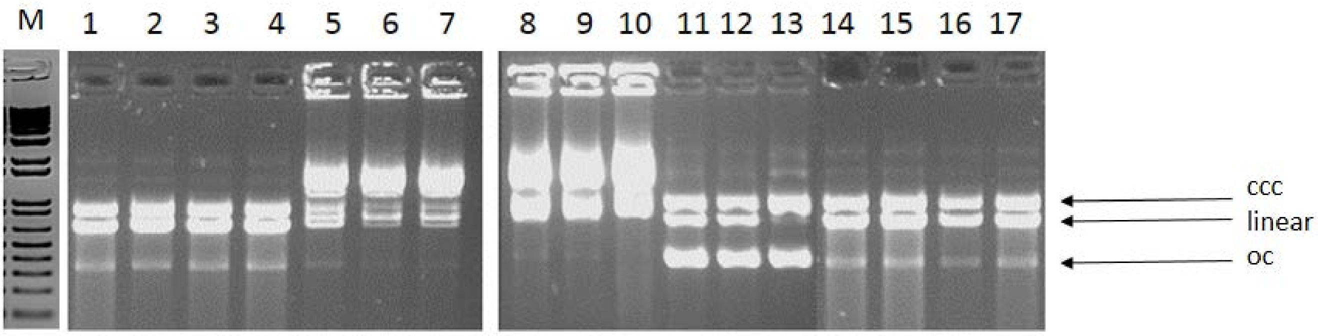

Figure S2. An example of an agarose gel electrophoresis separation of isolated plasmids DNA on R4 strains modified with selected lactones from 1-17 as described in Table 1

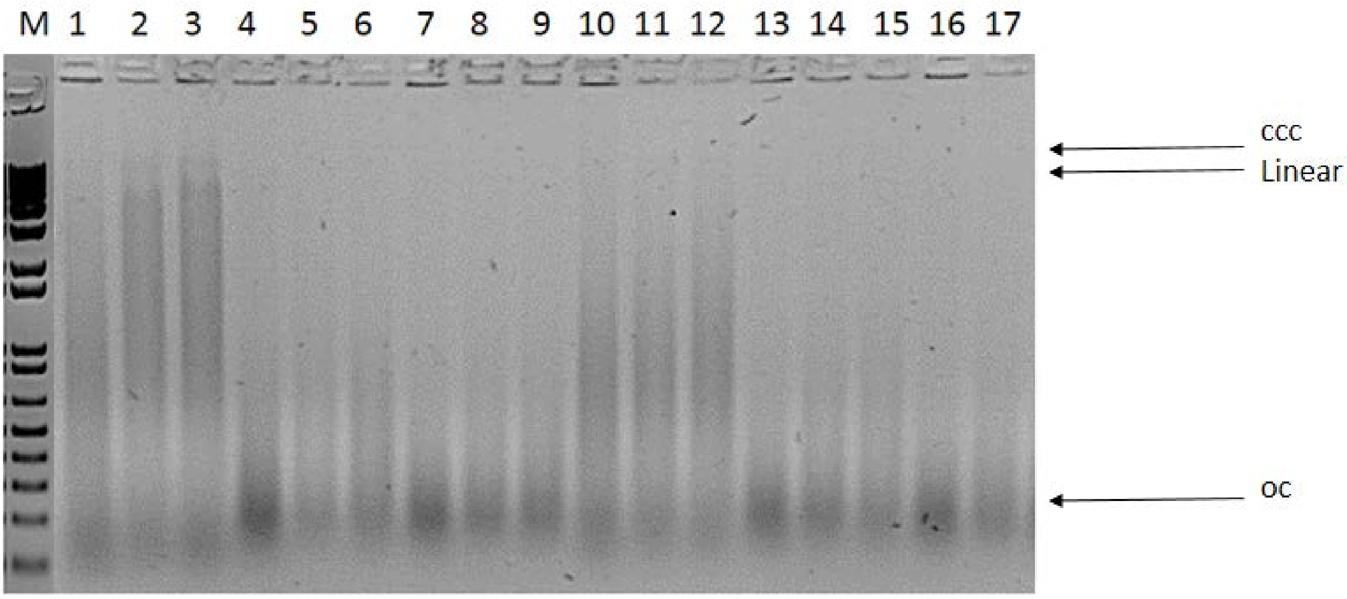

Figure S3. An example of an agarose gel electrophoresis separation of isolated plasmids DNA on R4 strains modified with selected lactones from 1-17 as described in Table 1, digested with repair Fpg protein.

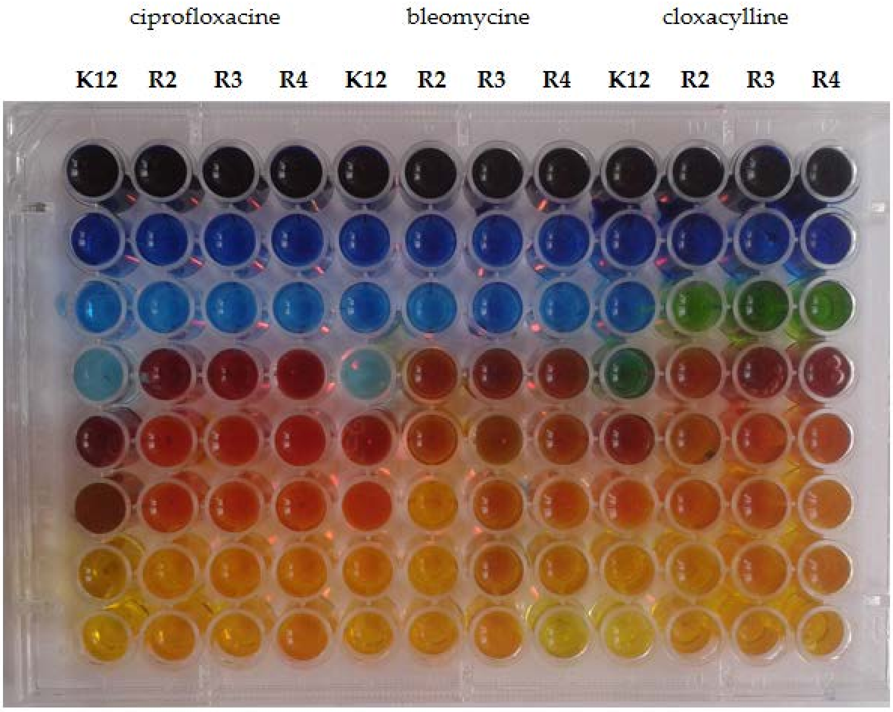

Figure S4. Examples of MIC with different strains K12, R2, R3, R4 of studied antibiotics with ciprofloxacin, bleomycin, cloxacillin.

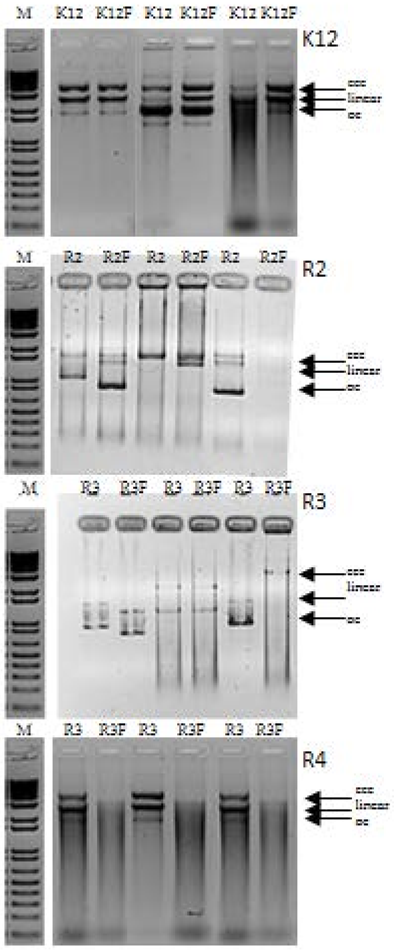

Figure S5. An example of an agarose gel electrophoresis separation of isolated plasmids DNA from K12,R2, R4 and R4 strains modified with antibiotics : ciprofloxacine, bleomycine and cloxacilline digested (or not) with repair enzymes Fpg.
